# Revisiting the Relationship Between Suicide and Unemployment in Mexico: Evidence From Linear and Non-linear Co-integration

**DOI:** 10.3389/fpubh.2020.00060

**Published:** 2020-03-04

**Authors:** Mei-Chih Wang, Feng-Li Lin, Hsiu-Hui Su, Pao Lan Kuo

**Affiliations:** ^1^Department of Finance, Providence University, Taichung, Taiwan; ^2^Department of Accounting, Chaoyang University of Technology, Taichung, Taiwan; ^3^Department of Applied English, Chaoyang University of Technology, Taichung, Taiwan; ^4^Department of Finance, Tamkang University, New Taipei City, Taiwan

**Keywords:** unemployment rate, suicide rate, economic growth, bootstrap ARDL bound test, granger causality test, C3: I1; J6

## Abstract

This study attempts to investigate if suicide is interlinked with unemployment in Mexico by making use of a recently developed Bootstrap ARDL bound test over the years of 1981–2016. To avoid omitting variable bias, we use economic growth rate as a control variable. The empirical results indicate that no co-integration among these three variables and there is a positively bidirectional causality between suicide rate and the unemployment rate. This study will showcase that the economic growth rate negatively affects unemployment rate and unidirectional Granger causality running from economic growth rate to the unemployment rate in Mexico. The findings presented in this study could provide with valuable information for society and health policy makers to formulate the policies on suicide prevention in Mexico.

## Introduction

From 2000 to 2011, about 54,000 suicides (i.e., 19.3%; about one in five) were reported a year worldwide where an estimated 233,000 suicides each year have been uncovered in the 63 countries, including third-world countries such as Mexico and Brazil as well as some first-world countries namely Denmark, Ireland, Poland, Russia, Greece, Spain, UK, Egypt, South Africa, US, Australia, Japan; Hong Kong, etc., These suicide cases were shown to be a result of unemployment (1). Moreover, the suicide rate has doubled to 4.7 per 100,000 people since 1990. Suicide occurs throughout the world and is often influenced or caused by different aspects such as religions, genders, cultures, and classes.

Unemployment is currently viewed by many countries as a crucial social issue that gives birth to suicide and this notion has been reflected in numerous comprehensive literatures. This phenomenon has been well-studied by different people from different regions. For instance, Gerdtham and Johannesson (2) studied about suicide rates in Swedish; Ruhm (3) and Neumayer (4) analyzed the occurrences of suicide in Germany; Webb et al. (5) and Yang and Lester (6) made use of research findings from the U.S., Kuroki (7) use Japanese data, Corcoran and Arensman (8) used information from Ireland, Barr et al. (9) used British data, Mattei and Pistoresi (10) used Italian data. To make the literature more comprehensive, cross-country data have also been used by Lin (11) who adopted research statistics from Asia. Breuer (12) and Reeves et al. (13) used European Union data, Noh (14) with the OECD-countries data. To better understand this research, it is essential to highlight that some studies only examined austerity, otherwise known as the effect of economic generosity, policies on suicide (10, 15, 16), whereas other studies reviewed the linkage between business cycles and suicide rates (1, 3, 12, 17–20). Another aspect that can also have an influence on suicide is economic fluctuations. The socio-economists and sociologists have exerted the effects of a probable asymmetric suicide cycle on the relationship between unemployment and suicide. For instance, Wu and Cheng (18) presented an asymmetric suicide cycles for men and working-age groups in the US. Chang and Chen (21) and Lin and Chen (22) suggested that suicide rates are pro-cyclical corresponding to the changes in the unemployment rate in the US. Mattei and Pistoresi (10) found a continuing effect deep-rooted up to 18 years in Italy with a 1% increase in long-term unemployment add up to the suicide rate by 0.83%.

Mexico is currently standing in the 11th place in terms of being the world's largest economy when measuring its GDP and purchasing power disparity. At the same time, the gap between the rich and the poor in Mexico is also the highest among the OECD (Organization for Economic Co-operation and Development) countries (23). According to the National Institute of Statistics, Geography and Informatics (NISGI), it has estimated a figure of two million adolescents between the ages of 14 and 19 to neither be studying or working. This group of people represents 13.4% of the Mexican population (24). On the other hand, the Mexican Institute of Youth (MIY) evaluated that 16 and 6% of youth between the ages of 15 and 19 as well as the ages of 12–14, respectively, are not undergoing any form of education, employment or professional training (NEET) despite the fact that mandatory education is provided to the citizens of the country up until the age of 15 (25). Thirteen point four percent of the Mexican who are categorized as NEET are socially excluded and left unsupervised. The lack of government responsibility in terms of meeting their societal expectations can potentially lead to various behavioral issues such as loneliness, dissatisfaction, abuse of substance and finally, suicide. According to the figures provided by the World Health Organization (WHO), Mexico's suicide rate was ranked in the 76th on a list of 109 countries (26). Moreover, its relevant annual suicide rate is 5.2 (male-8.2, female-2.3) per 100,000 people in 2016 (27). Investigating and examining the cause of suicide among citizens in Mexico is of immediate and crucial importance to Mexico's country, society as well as policy makers.

Up to now, research on different causes of Mexico's suicide is somewhat limited in the literature. Former studies that attempted to understand if unemployment can either increase or decrease suicide rates only brought about mixed results. These said studies also failed to use the asymmetric Granger causal linkage to inspect the relationship between suicide rates and unemployment in Mexico. The primary aim of this research paper is to evaluate Mexico's trivariate relationship between unemployment, suicide and economic growth from 1981 to 2016. We have been using the recently developed Bootstrap autoregressive distributed lags (NARDL) bound model by McNown et al. (28) as it has shown its ability to simultaneously look for the responses of both long and short-run asymmetric effects of unemployment on the suicide rates in Mexico. The main contribution in this study is to scrutinize the asymmetric Granger causal correlation between unemployment and suicide rates in Mexico beyond those of the existing research on the socioeconomic determinants of suicide. Examining and being aware of the suicidal behavior is crucially important to society and health policy makers in Mexico. Since Pesaran et al. (29) established their ARDL bound test, prior researches using ARDL econometric methodology to study the potential asymmetric effects of unemployment on suicide rates have gone through various types of transformation. For instance, Wu and Cheng (18) researched an asymmetric suicide cycle test of the US via a single ARDL test. Chang and Chen (21) employed the non-linear autoregressive distributed lags (NARDL) model suggested by Shin et al. (30) tried to understand how the unemployment of citizens affects suicide rates in the U.S. As of recently, McNown et al. (28) have taken a step further and altered this test via bootstrap techniques and this newer version of the bootstrap ARDL bound test has depicted numerous types of superiority over the older version of the ARDL bound test of Pesaran et al. (29). The newly developed bootstrap ARDL bound model has shown its capability to further enhance the bootstrap examining methods, as recommended by McNown et al. (28). It is also crucial to note that several new and appealing features stand out when examining the newer test with that of the conventional ARDL bound test of Pesaran et al. (29).

First, the Bootstrap ARDL bound test showcases various evidence of indigeneity issues that are said to carry minor influences on the power properties of ARDL bounds. The bound testing framework makes use of the asymptotic critical values from the Monte Carlo simulations. Furthermore, the bootstrap test has shown the capability to perform better if the test has rewired the size and power properties of the resampling procedure to the most accurate and appropriate ones. Secondly, the bootstrap method carries the extra advantage of excluding the chances of improper inferences. At last, with the correct critical value received from the process, the bootstrap procedure can show the extension of the said testing framework for an alternative degenerate case. There may be a non-linear response to both the rates of suicide and unemployment owing to the existence of economic fluctuation. To identify reasons why there might be a non-linear linkage between suicide and unemployment into consideration, this study made use of a non-parametric (31) score and rank which has the ability to test unemployment and suicide rates in both linear and non-linear frameworks if they are interlinked. If these variables are indeed interdependent, we employ the score test to understand if the integration either linear and non-linear. Thus, the results obtained via the bootstrap ARDL test along with the score test will offer a complete picture of the relationship between suicide and unemployment rates than the previously existing studies have. These empirical results indicate that there is a positively bidirectional causality between suicide rate and unemployment rate in Mexico, suggesting among those to be unemployed have an increased risk of suicide (3, 32). Similarly, the risk of suicide increases just as unemployment rises as they are shown not to be mutually exclusive (1, 2, 7–10, 12, 13, 33–35). Studying and knowing the suicidal behavior is of crucial importance to society and health policy makers. Via making advantageous policies for labor market policies, we can simultaneously increase the effectiveness of the market to produce additional jobs and decrease the unemployment rate, Mexico's governments could help decrease suicide risks. Finally, the limitation of this study is to analyze the relationship between unemployment, suicide and economic growth. However, other factors (social, cultural and medical aspects, etc.) may have some effect on suicides.

This study is organized in the following way. The second section of this study provides a deep and comprehensive literature review while the third section discusses the data used. Section Empirical Results presents the various results, namely empirical results and finally, the last section aims to offer different policy implications and hopes to conclude this study.

## Literature Review

We can see that business cycles tend to have a different influence on suicide rates. For instance, Viren (17) examines the linkage between business cycles and suicide in Finland during the years 1878 and 1994. The results show that suicide increased just as the age of the citizens increased and that it is inversely linked to GDP growth, unemployment and bankruptcies. Luo et al. (19) reviewed the associations of general and age-specific suicide rates as shown in the business cycles from the year 1928 to 2007 in the United States. Graphical analyses have illustrated that the general rate of suicide commonly increased during times of recessions and dropped during times of expansions. dos Santos et al. (20) examined the ways numerous social and economic aspects of society affect suicide in Portugal from 1910 to 2013. They found a strong link between the decline of growth rate as well as the rise in suicide rates for the general country. In terms of this, we can assert that suicide rates amongst men are undeniably affected by economic changes, whereas the suicide rates in women tend to be a response to a change in the political and economic environment.

Some studies specially examined the influence of economic generosity (austerity) policies on suicide. Korhonen et al. (15) used an economic model that showcases the changes in consumer welfare and how it constitutes a propagation mechanism to study the determiners of aggregate suicide in 15 countries from the year 1960–2010. The innovative feature of their findings is the theoretical model forecasting that the aggregate suicide rates are related to an economic downturn index which in turn has its roots in private consumerism. Chan et al. (16) evaluated the influence of the social welfare system on the relationship between unemployment and suicide via monthly data from 1991/1 to 2012/12 in Taiwan. Their findings suggested that the positive suicide-unemployment nexus is offset, especially after the passing of welfare policies by authorities in hopes to take care of people in the minority who are facing financial problems.

According to the comprehensive literature, unemployment is said to be a big factor to cause suicidal behavior. For instance, Yang and Lester (6) argue via long-term time-series analysis of the association between suicide and unemployment seemingly indicates to be powerful in the United States but almost non-existent in the remainder of the countries. By making use of a large individual data set comprising of almost 30,000 people in Sweden, Gerdtham and Johannesson (2) discovered that unemployment significantly increases the risk of suicides. Webb et al. (5) found when unemployment precedes suicide by 1 year, a decent and observable relationship between unemployment and suicide was also found for Whites of both gender, especially for males in the United States from 1929 to 1992.

On the other hand, Kuroki (7) showed that a rise in unemployment also greatly caused an increase in the suicide rate of men from 1985 to 2007 in Japan. The analysis based on suicide rates that are age-specific illustrates that unemployment chiefly influences working men from the ages of 35 to 64 but primary has the biggest impact on men who are ff to 64 years old. By making use of the Poisson regression, Corcoran and Arensman (8) evaluated the status of employment as well as the risk of suicide as depicted in Ireland from 1996 to 2006. This is a decade marked by an economic-boom which is commonly known as the Celtic Tiger. The unemployment rate in this period marked a sharp drop in the unemployment rate from 12% in 1996 to 4% in 2000, a level that remained constant until 2006. Their outcomes suggested that compared to employment, unemployment was linked with a 2–3-time rose risk of male suicide and undecided death, but commonly a 4–6-time rose risk in women. Unemployment was related to the great rose risk of suicide and uncertain death when its level was lower (2001~2006) than in the period of declining unemployment (1996~2000). Barr et al. (9) evaluated the British data and found that the number of employed men increased by 10%, which is a 1.4% increase in male suicides. Their findings show that a considerable percentage of the increase in suicides among men from 2008 to 2010 can be attributed and caused by the recession and hence the rising of unemployment. Milner et al. (35) conducted a systematic review of 16 studies, measured unemployment duration in different ways, out of a possible 10,358 articles found that long-term unemployment was related to a greater incidence of risk. These findings of the meta-analysis indicate that risk is the largest in the first 5 years, and continues to persist at an elevated level, though the level is lower than it was 16 years after unemployment. Reeves et al. (13) evaluated the fluctuations in suicide rates in 20 European Union (EU) countries between 1981 and 2011. Their results, modified for previously existing trends and country-fixed effects models, display that each percentage point increase in male unemployment causes a 0.94% increase in male suicides. Breuer (12) employed a series of data set from 275 regions, accumulated from 29 European countries from 1999 to 2010. The results show that unemployment indeed has a hugely positive effect on suicide. Besides, economic growth in real-time has shown to negatively influence the rate of workingmen. Nordt et al. (1) studied the random coefficient models to evaluate suicide, population, and economy for 63 countries such as Mexico, Brazil, Denmark, Ireland, Poland, Russia, Greece, Spain, UK, Egypt, South Africa, US, Australia, Japan; Hong Kong, etc. between 2000 and 2011 across four regions. Their results suggested that the relevant danger of suicide that is linked with unemployment was uplifted by 20~30% or so. Suicides that have been caused by unemployment illustrated a nine-fold higher number of deaths that those that were a result of an economic crisis. Recently, by means of co-integration techniques, Mattei and Pistoresi (10) identified a long-term connection between the suicide rate and unemployment in Italy. As little as a 1% increase in long-term unemployment has shown to immediately raise the suicide rate by 0.83%. This long-term influence has the ability to last and persist 18 years after fruition. Public unemployment expenditure may lessen this association: when its yearly growth rate is higher than 0.18%, no unemployment effect is detectable on suicide.

In contrast, some studies show divergent results. Neumayer (4) has provided evidence on the estimation of aggregate German data by analyzing the fixed effects. He points out that employment rates and the rates of suicide in both men and women are negatively linked. A US micro health panel research results of Ruhm (3) depicted the recessions to have lower rates of mortality, even though suicides continued to increase over the years. Lin (11) highlights that suicide moves counter-cyclically as indicated by the two panel research data sets acquired from Taiwan and other countries. Additionally, to better understand the fixed effects approach, cross-country panel tended to eliminate variations that were cross-sectional, but simultaneously adopt variations that were linked with time-series for OECD. Noh (14) demonstrates that the implications of the results of unemployment are positively influenced by countries that have a population of people who have a higher income. There is a negative impact of unemployment on suicide has only been observed for countries that have citizens who are earning at a lower income.

It's of great importance to mention that the obscure affiliation between suicide and unemployment may be the product of economic fluctuation which in turn may give rise to an asymmetric effect on the rates of suicide. This asymmetric result of fluctuation has been well analyzed for broader literature. For instance, Lester (36) evaluated the curvilinear (asymmetric) impact of economic fluctuation on suicide and incorporate the interplay of both economic as well as sociological variables on suicide in the U.S. Wu and Cheng (18) studied various asymmetric suicide cycles test of American time-series data from 1951 to 2005. They offered a confirmation that asymmetric suicide cycles are indeed important for men in the workforce. However, their study cannot emphasize the same notion for women as well as the people who are not in the workforce. Using the ARDL approach of co-integration in Japan from 1957 to 2009, Andrés et al. (37) showed that a handful of sociological factors such as fertility and divorce should also be evaluated and at times had a stronger impact on suicide rates than economic factors like GDP and unemployment for women. Chang and Chen (21) employed the linear and non-linear ARDL integration methods to study the possible symmetric and asymmetric responses of suicide rates to unemployment rates in the US from the years 1928–2013. Their findings showed that economic expansion in society tends to have a bigger impact on lowering the suicide rates than economic recession on increasing it for people who were above the age of 45. Phiri and Mukuka (38) used the ARDL model to analyze the assimilation between unemployment and suicide in South Africa from 1996 to 2015. Their findings show that, contrary to the evidence illustrated by other countries, unemployment is not primarily dependent on suicide rates. The same cannot be said for citizens who are beyond the age of 75. Lin and Chen (22) employed the newly matured causality test that generalized the impulse-response method to prove the linkage between suicide and unemployment in the U.S. from 1928 to 2013. Their research data shows that there exists an unbalanced effect of unemployment on suicide.

Nonetheless, existing studies have researched the linkage between business cycles and suicide, the effect of economic policies and finally, the influence of unemployment (1, 2, 4, 7–17, 19, 20, 33–35). However, up till now, none of them examined the asymmetric Granger causal association between unemployment and suicide rates in the Mexico. Therefore, through bootstrap ARDL test accompanied by score test, this study will propose a comprehensive picture of the relationship between suicide and unemployment rates than the previous studies have presented.

## Data and Methodology

The leading ambition of this research study is to analyze and investigate the relationship between the suicide rate and the unemployment rate in Mexico. We tried to investigate them at the same time, adding the economic growth rate from 1981 to 2016. Furthermore, our empirical analysis obtained its yearly data that were obtained from the International Monetary Fund (IMF): World Economic Outlook. Each of these variables has been calculated by the way of the sequential difference of the natural logarithm of the variables before the econometric analysis.

The primary intention of this study is in two-folds: firstly, this paper will implement the bootstrap methodology developed by McNown et al. (28). It is the methodology that is derived from the ARDL co-integration test so that it can explore the linear causality, Also, due to the tiny sample of the research data, we made use of the bootstrap methodology to accurately assess the final products of the study. Secondly, we employed the non-linear Brietung's (31) supposed Score Tests to fully evaluate the long term causal link between suicide, unemployment and economic growth.

### ARDL Bootsreap Test (28)

By following the footsteps of McNown et al. (28), we build a preliminary equation to completely understand suicide and unemployment as in (1):

(1) $$\begin{array}{l} \Delta unemployment_{t}=\;c+\text{Ø}employment_{t-1}+{\text{Ø}suicide}_{t}\\ \;+\overset{p-1}{\underset{i=1}{\sum }}\lambda_{i\;}\Delta unemployment_{t-i}+\overset{q-1}{\underset{j=1}{\sum }}\lambda_{j}{\Delta suicide}_{t-j}\\ \;+\overset{s}{\underset{l=1}{\sum }}z_{l}D_{t,l}+\varepsilon_{t} \end{array}$$

(2) $$\begin{array}{l} \Delta suicide_{t}=\;c+\;\gamma unemployment_{t-1}+\gamma suicide_{t}\\ \;+\overset{p-1}{\underset{i=1}{\sum }}\delta_{i}\Delta unemployment_{t-i}+\overset{q-1}{\underset{j=1}{\sum }}\delta_{j}{\Delta suicide}_{t-j}\\ \;+\overset{s}{\underset{k=1}{\sum }}\omega_{l}D_{t,k}+\varepsilon_{t}\\ \;D_{t,l}\;D_{t,k}:dummy\;variables. \end{array}$$

From this, we determine the structural breaks for both Equations (1) and (2) by numerous structural break tests advised by Bai and Perron (39). The empirical results are shown in **Table 2**, e.g., the specific structural break dates of suicide rate and unemployment rate are 1986, 1994, and 2009. Adding GDP as a control variable, the specific structural break dates are 2001, 2009, and 2014.

We use the Bootstrap residuals to get all observations, then to estimate the ARDL model, and finally, we make use of bootstrap F test and t statistics. Then, we contrast the bootstrap distribution to determine the crucial figures and values.

Given that, we can also test the relationship between these two variables with the null hypothesis that is based on the two Equations (1) and (2).

$$\begin{array}{l} H_{0}{:\delta }_{2,i}=0\text{ for Equation }\left(1\right)\text{ and }H_{0}{\text{Ø}}_{1,i}=0\text{ for Equation }\left(2\right). \end{array}$$

Under the null hypothesis, *H*_0_:δ_2,*i*_ = 0 indicates that unemployment does not cause suicide; *H*_0_∅_1,*i*_ = 0 indicates a suicide rate does not affect the unemployment rate. We can utilize the Wald test to restrict the two coefficients.

McNown et al. (28) application of the bootstrap methodology of the ARDL tests of assimilation asserted that the aforementioned tests have the correct size and reasonable power properties. It is critical to emphasize that the adoption of all the tests is of importance so as to allow the cases to be identified as co-integration, non-co-integration, and degenerate cases, as described by Pesaran et al. (29). By fulling understanding McNown et al. (28), we can define the two degenerate cases by the following means:

(i) Degenerate case #1 occurs when the *F*-test and the *t*-test on the lagged independent variable are important, yet the *t*-test on the lagged dependent variable is insignificant.
(ii) Degenerate case #2 occurs when the *F*-test and the *t*-test on the lagged dependent are important, but the lagged independent variables are insignificant.

The critical value of the second case has been presented by Pesaran et al. (29) but not for the first case. The assimilation sequence of the dependent variable must be I (1) but the unit root tests have shown to be infamous as they tend to have low-power (40). The Bootstrap ARDL test tackles this issue by making use of supplementary examinations to better evaluate the coefficients that are presented for the lagged independent variables. That is the reason why the advantage of bootstrap ARDL is that utilizing asymptotic critical values of the simulation can present little effect on the power properties and the size of the ARDL test. Moreover, this bootstrap test tends to perform with more superiority over the asymptotic test when the resampling procedure is conducted properly. In addition, the Bootstrap process has the added benefit of removing the chances of observing inferences that are inconclusive. At last, McNown et al. (28) also showcased an expansion of the ARDL testing for another degenerate case by making use of the crucial values created from the process. Thus, the Bootstrap ARDL test gives a higher level of insight provides a better insight into the integration of the sequences in the model.

### Score Test for Non-linearilty

Brietung (31) suggests a score test statistic *T* · *R*^2^ to fully understand if integration is either non-linear or linear, then compute the following regression:

(3) $$\begin{array}{r} {\tilde{\propto }}_{t}=b_{0}+bx_{t}+b_{2}R\left(x_{t}\right)+e_{t} \end{array}$$

Where *T* is the sample size, *R*^2^ is the coefficient to determine regression (1), and ${\tilde{\propto }}_{t}$ = *y*_*t*−_(ã_0_ + ã_1_*x*_*t*_), where ã_0_ and ã_1_ are the squares estimates of a regression of y_t_ on a constant and x_t_. Because of this, we can assume that u_t_ is a zero-mean noise and x_t_ is exogenous, the score test figure *T* · *R*^2^ is asymptotically Chi-squared (χ^2^) and is distributed with only a one degree of freedom. The hypothesis of linear co-integration, b_2_ = 0, can be dismissed in favor of non-linear co-integration if they *T* · *R*^2^ were to outpace the χ^2^ critical value. Though, Brietung (31) has highlighted in numerous cases that x_t_ is internal. Brietung (31) also advised to take in the co-integration regression because of Stock and Watson (41) for modification as by doing so the infinite sum will be correctly depicted as follows:

(4) $$\begin{array}{r} y_{t}=\alpha_{0}+\overset{\infty }{\underset{j=1}{\sum }}\alpha_{j}y_{t-j}+\beta_{1}x_{t}+\overset{\infty }{\underset{j=-\infty }{\sum }}\gamma_{j}\Delta x_{t-j}+\varepsilon_{t} \end{array}$$

The least squares estimated residual ε_t_ will be then lessened on Equation (3) and R(x_t_). This will lead to the linear co-integration relationship of the null hypothesis ending up with *T* · *R*^2^, where *R*^2^ is the coefficient to represent regression (4). This is also asymptotically Chi-squared (χ^2^) and like before, is similarly scattered with one degree of freedom. The simulations of Monte Carlo by Brietung (31) are indicative of how the wide range of that non-linear models performs better than their parametric competitors.

### Kruse (42) Non-linear Unit Root Test

We applied the Kruse (42) non-linear unit root test, which is an extension of Kapetanios et al. (43) one, that assumes that in the first specified model along (5), the location parameter, δ ought not to be zero.

(5) $$\begin{array}{r} \Delta x_{t}=\alpha x_{t-1}+\beta x_{t-1}\left[\left(1-\exp\left(-\delta x_{t-d}^{2}\right)\right)\right]+\varepsilon_{t}\; \end{array}$$

If *x*_*t*_ is the series, then ε_*t*_is the error term as it has to appease the classical assumptions. Therefore, it is also the transition parameter. Since the parameter is unable to be classified under the null, Taylor approximation would be used as:

$1-\exp\left(-\delta x_{t-d}^{2}\right)$ by continuing with the auxiliary regression:

(6) $$\begin{array}{r} \Delta x_{t}=\delta x_{t-1}^{3}\overset{j}{\underset{i-1}{\sum }}\theta_{i}x_{t-i}+\epsilon_{t}\; \end{array}$$

This test has the main advantage of using zero as the location parameter in its function. The results that can be seen above emphasize that the null hypothesis of the unemployment rate, suicide rate and the economic development of Mexico is represented by the non-linear unit root e, and ought to be dismissed at least at the 1 percent level.

## Empirical Results

### Results From the Unit Root Tests

From Table 1, we should first test the stationary of unemployment rate, suicide rates, and the economic growth rate. All variables are skewed to the left, which means they lack symmetry in the data distribution. The kurtosis peak of GDP is higher and more prominent. This reflects that the data is heavy-tailed. We consider suicide rates and unemployment rates can be non-linear, so we employed augmented Dickey and Fuller (44) and the Kruse (42). ADF tests were not able to dismiss the null of a unit root for the unemployment rate and the suicide rate in Mexico at level, however, all are ignored the null of a unit root at first difference. The Kruse test yields reject the null of a unit root. Our results signify that unemployment rates, suicide rates and economic growth in Mexico are all random processes.

**Table 1:** Summary statistics and unit root test.

	**GDP**	**Unemployment**	**Suicide**
Mean	2.410957	3.930537	4.105556
Median	2.851100	3.806163	4.200000
Maximum	8.525607	6.228353	5.500000
Minimum	−6.291231	0.900000	2.300000
Std. Dev.	3.202466	1.152522	0.837665
Skewness	−0.908875	−0.032776	−0.308184
Kurtosis	3.909154	2.988767	2.313113
Jarque–Bera	6.196166	0.006635	1.277585
ADF (level)	−6.545 (0.000)^***^	−1.481 (0.531)	−1.481 (0.531)
ADF(1_st_ differece)	−9.428 (0.000)^***^	−9.545 (0.000)^***^	−9.545 (0.000)^***^
Kruse tau test	24.180^***^	21.346^***^	49.036^***^

*** *denotes the significant levels at 1%*.

## Results

Table 2 reports, structural break dates of suicide rate and unemployment rate are 1986, 1994, and 2009, especially 1994 and 2009 regarding the financial crisis. Adding GDP as a control variable, structural break dates of suicide rate and unemployment rate are 2001, 2009, and 2014, which are all global financial crisis. The empirical co-integrating results based on the Bootstrap ARDL approach. In terms of the bootstrap *F* statistic, *t* statistics included lagged dependent variables and independent variables; we found no significant co-integration that would exist in the unemployment rate and suicide rate regardless of the economic growth rate (Table 2).

**Table 2:** Co-integration analysis.

**DV |IV**	**Dummy variable**	F^*^	***F***	T^*^dep	**T_**dep**_**	**${\text{F}}_{\text{indep}}^{\text{8}}$**	**F_**indep**_**	**Result**
UN|SU	D86 D94 D09	5.7578	−3.1855	3.17739	7.2886	−3.8111	0.9043	No co-integration
SU|UN	D86 D94 D09	4.8204	−2.7301	3.8760	4.9133	−3.3878	0.6640	No co-integration
UN|SU,GDP	D01 D09 D14	5.7578	−3.1855	3.17739	7.2886	−3.8111	0.9043	No co-integration
SU|UN,GDP	D01 D09 D14	5.7578	−3.1855	3.17739	7.2886	−3.8111	0.9043	No co-integration

The prime lag order based on Schwarz Bayesian Criterion (SBC). F is the F-statistic for the coefficients of Y_t−1_, X_t−1_; Tdep denotes the t-statistics for the dependent variable, Findep denotes the F-statistics for the independent variable. F^*^, Tdep^*^ and Tindep^*^ are the critical values at 10% significance level, generated from the bootstrap program. Dummy variables are to capture any economics shocks. D02 means 1 for year 2012, other years are 0.

Therefore, we tried to use the score test of Breitung (31) to know if the linkage is direct or indirect, then we discovered that the non-linear co-integrating relationship exists in Table 3. It has been observed that the null hypothesis of direct linkage was rejected in all scenarios. The score test showcases that an indirect co-integrating relationship is present between the unemployment rates, suicide rates and GDP for Mexico, suggesting suicide rates and unemployment rates associated with increases or decreases in GDP in the long term.

**Table 3:** Score tests for non-linearity.

**Country**	**Score test (without GDP)**	**Score test (with GDP)**
	**T ⊕ *R*^2^**	**T ⊕ *R*^2^**
Unemployment	5.2536^**^	5.3939^***^
Suicide	4.1381^**^	5.9056^***^
GDP	3.3615^**^	8.84817^***^
Critical value (%)		
10	2.71	
5	3.84	
1	6.63	

a The bivariate rank test has been adjusted for autocorrelation. The null hypothesis of the test shows that no co-integration can be seen the exchange rate and relative price; the alternative hypothesis is otherwise. The null hypothesis is dismissed if the crucial number exceeds the test statistic.

b ^**^ and ^***^ denote significance at the 5% and 1% levels, respectively.

**Figure 1:**
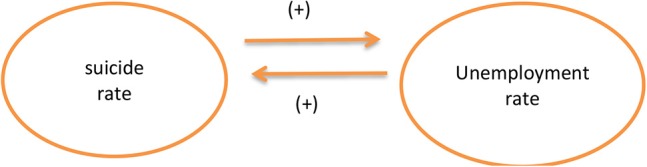
Causal link between suicide rate and unemployment rate.

To further investigate the causality depicted in Tables 4, 5, we adopted the Wald test to reveal the link to better understand the two variables. In Table 4 and Figure 1, we found a positive relationship between the unemployment rates and suicide rates regardless of economic growth rate, suggesting an increased risk of suicide among those who become unemployed (32), likewise, the association between an increase in the unemployment rate as well as suicide rate has confirmed to be accurate (33, 34). The empirical results presented in our study mirror and reflect the ones presented by Gerdtham and Johannesson (2), Ruhm (3) and Neumayer (4), Webb et al. (5), Yang and Lester (6), Kuroki (7), Corcoran and Arensman (8), Barr et al. (9), and Mattei and Pistoresi (10), there is indeed a dual-link between unemployment rates and suicide rates both in the short run. Yet, it is also important to note that our empirical findings are inconsistent with those of Neumyer (45), Andrés (46), and Kim and Cho (47). Due to the advanced and comprehensive techniques and methods applied in our study, we believe the findings presented in our research to be more reliable and dependable.

**Table 4:** Granger-causality analysis based on bootstrap ARDL models.

**Country**	**Δ *UN equation***	**Δ *SU equation***
	***F* or *t* statistic (*p-*value)**	***F* or *t* statistic (*p-*value)**
Δ un_t_, *un*_*t*−1_,	n.a.	3.1936^***^(0.006) (+)
Δ su_t_, *su*_*t*−1_	29.491^***^ (0.000) (+)	n.a.

*** *denotes 1% significant level*.

**Table 5:** Granger-causality analysis based on bootstrap ARDL models.

**Country**	**Δ *UN equation***	**Δ *SU equation***
	***F* or *t* statistic (*p-*value)**	***F* or *t* statistic (*p-*value)**
Δ un_t_, *un*_*t*−1_,	n.a.	3.161^*^ (0.057) (+)
Δ su_t_, *su*_*t*−1_	6.394^***^ (0.007) (+)	n.a.
Δgdp_t_, *gdp*_*t*−1_	6.295^***^ (0.00 8) (–)	0.2186 (0.684) (–)

*** and ^*^ denote 10 and 1% significant level, respectively.

**Figure 2:**
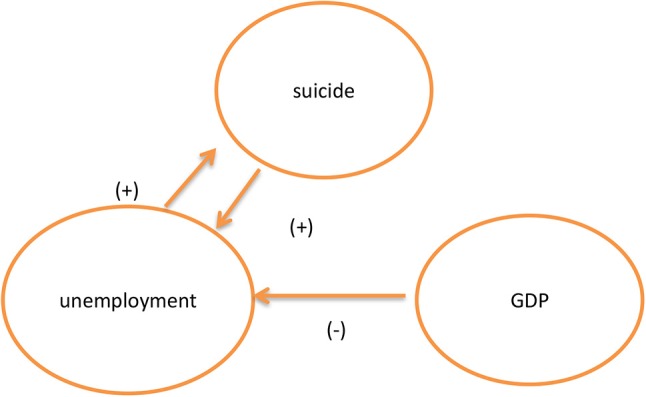
Causal link among GDP, suicide rate, and unemployment rate.

Interesting is that we find economic growth rates are a negative effect on unemployment rates after adding to economic growth rates, namely, the economy is worse, the unemployment rates are up, and suicide rates are bound to rise as indicated in Table 5 and Figure 2. These results are inconsistent with those of Viren (17) and Luo et al. (19) who found that economic growth affects suicide rates. For Mexican policymakers, we suggest no matter what the economic growth is, the government improves job opportunities to reduce the suicide rate or improve social protection programs as well as active labor market programs which should aid in assisting the unemployed in finding work.

## Conclusion

The global suicide rate is steadily falling, from a high of 38 percent in 1994 to just 11 percent in 2018. However, the opposite is true for Mexico as suicide rates have been rising over the past 40 years, but little literature discusses the reason. From an empirical study, the paper's primary contribution is finding that the economic growth directly negatively affects the unemployment rate, at the same time, the unemployment rate and suicide rate are positive bidirectional causality in Mexico. Moreover, we find that adding the variable of economic growth rate as a control variable, unemployment rate, suicide rate, and the economic growth rate exists long relationship under non-linear conditions.

In this study, we employed the highly advanced bootstrap ARDL co-integration test for linearity and Score test for non-linearity to investigate the long-run relationship between unemployment rates, suicide rates and economic growth rates as a control variable. The empirical results say non-linear significant long-run co-integration exists. Furthermore, we examined the causality based on the ARDL approach. However, positive bidirectional causality is found between unemployment rates and suicide rates. Additionally, there is a unit negative effect from the economic growth to unemployment.

In conclusion, understanding the causal relationship between economic growth, unemployment rates and suicide rates is a crucial necessity to policymakers so that they can employ the appropriate unemployment strategies to reduce suicide rates especially economic recession results in higher suicide rates. The factual results as illustrated in our study have vital implications for the Mexican government.

## Data Availability Statement

The raw data supporting the conclusions of this article will be made available by the authors, without undue reservation, to any qualified researcher.

## Author Contributions

All authors listed have made a substantial, direct and intellectual contribution to the work, and approved it for publication.

### Conflict of Interest

The authors declare that the research was conducted in the absence of any commercial or financial relationships that could be construed as a potential conflict of interest.

## References

[1] Nordt C Warnke I Seifritz E Kawohl W Modelling suicide and unemployment: a longitudinal analysis covering 63 countries 2000-2011. Lancet Psychiatry. (2015) 2:239–45. 10.1016/S2215-0366(14)00118-7 26359902

[2] Gerdtham U-G Johannesson M. A note on the effect of unemployment on mortality. J Health Eco. (2003) 22:505–18. 10.1016/S0167-6296(03)00004-3 12683964

[3] Ruhm CJ Are recessions good for your health? Q J Eco. (2000) 115:617–50. 10.1162/003355300554872

[4] Neumayer E. Recessions lower (some) mortality rates: Evidence from Germany. Soc Sci Med. (2004) 58:1037–47. 10.1016/j.socscimed.2004.02.018 14723900

[5] Webb LD Glass G Metha A Cobb C Economic correlates of suicide in the United States (1929–1992): a time series analysis. Arch Suicide Res. (2002) 6:93–101. 10.1080/13811110208951167

[6] Yang B Lester D Suicide, homicide, and unemployment. Appl Eco Lett. (1995) 2:278–9. 10.1080/135048595357221

[7] Kuroki M The Suicide and unemployment in Japan: Evidence from municipal level suicide rates and age-specific suicide rates. J Soc Eco. (2010) 39:683–91. 10.1016/j.socec.2010.06.009

[8] Corcoran P Arensman E. Suicide and employment status during ireland's celtic tiger economy. Eur J Public Health. (2011) 21:209–14. 10.1093/eurpub/ckp236 20110275

[9] Barr B Taylor-Robinson D Scott-Samuel A McKee M Stuckler D Suicides associated with the 2008-2010 economic recession in England: time trend analysis. Br Med J. (2012) 345:5142 10.1136/bmj.e5142 PMC3419273 22893569

[10] Mattei G Pistoresi B. Unemployment and suicide in Italy: evidence of a long-run association mitigated by public unemployment spending. Eur J Health Eco. (2019) 20: 569–77. 10.1007/s10198-018-1018-7 30542937

[11] Lin SJ Unemployment and suicide: Panel data analyses. Soc Sci J. (2006) 43:727–32. 10.1016/j.soscij.2006.08.013

[12] Breuer C. Unemployment and suicide mortality: evidence from regional panel data in europe. Health Eco. (2015) 24:936–50. 10.1002/hec.3073 24934277

[13] Reeves A McKee M Gunnell D Chang SS Basu S Barr B. Economic shocks, resilience, and male suicides in the Great Recession: cross-national analysis of 20 EU countries. Eur J Public Health. (2015) 25:404–9. 10.1093/eurpub/cku168 25287115

[14] Noh YH Does unemployment increase suicide rates? The OECD panel evidence. J Eco Psychol. (2009) 30:575–82. 10.1016/j.joep.2009.04.003

[15] Korhonen M Puhakka M Viren M Economic hardship and suicides. Int J Soc Eco. (2017) 44:1348–60. 10.1108/IJSE-06-2016-0153

[16] Chan YS Liu TC Chen CS Peng YI A changing nexus between unemployment and suicide in Taiwan: before and after labor welfare improvement in the late 1990s. Soc Indicat Res. (2018) 140:333–46. 10.1007/s11205-017-1772-4

[17] Viren M Suicide and business cycles: finnish evidence. Appl Eco Lett. (1996) 3:737–8. 10.1080/135048596355781

[18] Wu WC Cheng HP. Symmetric mortality and asymmetric suicide cycles. Soc Sci Med. (2010) 70:1974–81. 10.1016/j.socscimed.2010.01.052 20378221

[19] Luo F Florence CS Quispe-Agnoli M Ouyang L Crosby AE. Impact of business cycles on US suicide rates 1928–2007. Am J Public Health. (2011) 101:1139–46. 10.2105/AJPH.2010.300010 21493938 PMC3093269

[20] dos Santos JP Tavares M Barros PP. More than just numbers: Suicide rates and the economic cycle in Portugal (1910–2013). SSM Popul Health. (2016) 2:14–23. 10.1016/j.ssmph.2015.11.004 29349124 PMC5757999

[21] Chang T Chen WY Revisiting the relationship between suicide and unemployment: evidence from linear and nonlinear co-integration. Eco Syst. (2017) 41:266–78. 10.1016/j.ecosys.2016.06.004

[22] Lin YH Chen WY Does unemployment have asymmetric effects on suicide rates? Evidence from the United States: 1928–2013. Eco Res Ekonom IstraŽ. (2018) 31:1404–17. 10.1080/1331677X.2018.1484788

[23] OECD (Organization for Economic Co-operation and Development) (2017). OECD Economic Surveys: Mexico.

[24] INEGI (Instituto Nacional de Estadisica Geografia e Informatica) Encuesta Nacional de Ocupacion y Empleo 2009. Available online at: http://www.inegi.org.mx/prod_serv/contenidos/espanol/bvinegi/productos/encuestas/hogares/enoe/enoe2009/ENOE_2009.pdf (accessed October 17, 2010).

[25] IMJUVEInstituto Mexicano de la Juventud) Jovenes Mexicanos: Encuesta Nacional de Juventud 2005. Available online at: http://cendoc.imjuventud.gob.mx/investigacion/docs/ENJ2005-TomoI.swf (accessed October 12, 2010).

[26] Mexico News Daily Suicides Up but the Rate Is Still Low. (2014)

[27] WHO (2016). Suicide Rates Data by Country. Available online at: http://apps.who.int/gho/data/node.main.MHSUICIDEASDR?lang=en

[28] McNown R Sam CY Goh SK Bootstrapping the autoregressive distributed lag test for co-integration. Appl Eco. (2018) 50:1509–21. 10.1080/00036846.2017.1366643

[29] Pesaran HM Shin Y Smith RJ Bounds testing approaches to the analysis of level relationships. J Appl Eco. (2001) 16:289–326. 10.1002/jae.616

[30] Shin Y Yu B Greenwood-Nimmo M Modelling Asymmetric Co-integration and Dynamic Multipliers in and Nonlinear ARDL Framework. Chapter 9. New York, NY: Springerlink (2014). 10.1007/978-1-4899-8008-3_9

[31] Breitung J Rank tests for nonlinear co-integration. J Bus Eco Stat. (2001) 19:331–40. 10.1198/073500101681019981

[32] Stack S. Suicide: a 15-year review of the sociological literature part I: cultural and economic factors. Suic Life Threat Behav. (2000) 30:145–62. 10.1111/j.1943-278X.2000.tb01073.x 10888055

[33] Economou A Nikolaou A Theodossiou I Are recessions harmful to health after all? Evid Eur Union J Eco Studies. (2008) 35:368–84. 10.1108/01443580810903536

[34] Stuckler D Basu S Suhrcke M Coutts A McKee M. The public health effect of economic crises and alternative policy responses in Europe: an empirical analysis. Lancet. (2009) 374:315–23. 10.1016/S0140-6736(09)61124-7 19589588

[35] Milner A Page A LaMontagne AD. Long-term unemployment and suicide: a systematic review and meta-analysis. PLoS ONE. (2013) 8:e51333. 10.1371/journal.pone.0051333 23341881 PMC3547020

[36] Lester BY Learning from Durkheim and beyond: the economy and suicide. Suic Life Threat Behav. (2001) 31:15–31. 10.1521/suli.31.1.15.21306 11326766

[37] Andrés AR Halicioglu F Yamamura E Socio-economic determinants of suicide in Japan. J Soc Eco. (2011) 40:723–31. 10.1016/j.socec.2011.08.002

[38] Phiri A Mukuka D “Does unemployment aggravate suicide rates in South Africa? Some empirical evidence,” Working Papers 1705, Department of Economics, Nelson Mandela University, revised Jul 2017. (2017).

[39] Bai J Perron P Computation and analysis of multiple structural change models. J Appl Eco. (2003) 18:1–22. 10.1002/jae.659

[40] Perron P The great crash, the oil price shock, and the unit root hypothesis. Econometrica. (1989) 57:1361–401.

[41] Stock JH Watson MW A simple estimator of co-integrating vectors in high order integrated systems. Econometrica. (1993) 61:783–820. 10.2307/2951763

[42] Kruse R A new unit root test against ESTAR based on a class of modified statistics. Statist Papers. (2011) 52:71–85. 10.1007/s00362-009-0204-1

[43] Kapetanios G Shin Y Snell A Testing for a unit root in the nonlinear STAR framework. J Eco. (2003) 112:359–79. 10.1016/S0304-4076(02)00202-6

[44] Dickey DA Fuller WA Distribution of the estimators for autoregressive time series with a unit root. J Am Stat Assoc. (1979) 74:427–31. 10.1080/01621459.1979.10482531

[45] Neumayer E Are socioeconomic factors valid determinants of suicide? Controlling for national cultures of suicide with fixed-effects estimation. Cross Cult Res. (2003) 37:307–29. 10.1177/1069397103253708

[46] Andrés AR Income inequality, unemployment, and suicide: A panel data analysis of 15 European countries. Appl Econ. (2005) 37:439–51. 10.1080/0003684042000295304

[47] Kim C Cho Y. Does unstable employment have an association with suicide rates among the young? Int J Environ Res Public Health. (2017) 14:470. 10.3390/ijerph14050470 28452940 PMC5451921

